# APTC-C-SA01: A Novel Bacteriophage Cocktail Targeting *Staphylococcus aureus* and MRSA Biofilms

**DOI:** 10.3390/ijms23116116

**Published:** 2022-05-30

**Authors:** Sha Liu, Karen Hon, George Spyro Bouras, Alkis James Psaltis, Keith Shearwin, Peter-John Wormald, Sarah Vreugde

**Affiliations:** 1 Department of Surgery-Otolaryngology Head and Neck Surgery, Basil Hetzel Institute for Translational Health Research, Central Adelaide Local Health Network, Woodville, SA 5011, Australia; sha.liu@adelaide.edu.au (S.L.); karen.hon@adelaide.edu.au (K.H.); george.bouras@adelaide.edu.au (G.S.B.); alkis.psaltis@adelaide.edu.au (A.J.P.); peterj.wormald@adelaide.edu.au (P.-J.W.); 2 Adelaide Medical School, The University of Adelaide, Adelaide, SA 5000, Australia; 3 School of Biological Sciences, The University of Adelaide, Adelaide, SA 5000, Australia; keith.shearwin@adelaide.edu.au

**Keywords:** *S. aureus*, antimicrobial, bacteriophage, biofilm, phage cocktail

## Abstract

The high infection and mortality rate of methicillin-resistant *Staphylococcus aureus* (MRSA) necessitates the urgent development of new treatment strategies. Bacteriophages (phages) have several advantages compared to antibiotics for the treatment of multi-drug-resistant bacterial infections, and thus provide a promising alternative to antibiotics. Here, *S. aureus* phages were isolated from patients and environmental sources. Phages were characterized for stability, morphology and genomic sequence and their bactericidal activity against the biofilm form of methicillin-susceptible *Staphylococcus aureus* (MSSA) and MRSA was investigated. Four *S. aureus* phages were isolated and tested against 51 MSSA and MRSA clinical isolates and reference strains. The phages had a broad host range of 82–94% individually and of >98% when combined and could significantly reduce the viability of *S. aureus* biofilms. The phages had a latent period of ≤20 min and burst size of >11 plaque forming units (PFU)/infected cell. Transmission electron microscopy (TEM) identified phages belonging to the family of *Myoviridae*. Genomic sequencing indicated the lytic nature of all four phages, with no identified resistance or virulence genes. The 4 phages showed a high complementarity with 49/51 strains (96%) sensitive to at least 2/4 phages tested. Furthermore, the frequency of bacteriophage insensitive mutant (BIM) generation was lower when the phages were combined into the phage cocktail APTC-C-SA01 than for bacteria exposed to each of the phages alone. In conclusion, APTC-C-SA01, containing four lytic *S. aureus* phages has the potential for further development as a treatment against MSSA and MRSA infections.

## 1. Introduction

The misuse and overuse of antibiotics greatly contributes to the rapid development of antimicrobial resistance, which has been described by the World Health Organization (WHO) as one of the top 10 global public health threats facing humanity [1,2,3]. Notably, the rise of the ‘superbug’, resistant to all or most commonly used antibiotics, has increased the mortality and morbidity rate among patients due to the lack of suitable treatments [1]. Such infections mainly affect patients who have been hospitalized long-term, often in intensive care units, patients who have been administered antimicrobial drugs long-term, or those who have undergone invasive treatment or with low immunity such as in the context of cancer. Those patients are often vulnerable to hospital-acquired superbug infections in the form of either respiratory infections or direct contact infections [4,5,6,7,8].

In 2017, the WHO listed several pathogens for which the development of novel antibiotics is urgently needed [9]. This list included *Staphylococcus aureus*, a commensal that colonizes more than 30% of American adults’ nasal cavities but can also cause infections in a range of severity and localizations [10]. These infections include local skin infections such as folliculitis, boils, and pustules, as well as abscesses, and can spread to bone tissue (osteomyelitis), lungs (Staphylococcal pneumonia), blood (bacteremia or sepsis), heart (infective endocarditis), and other organs [11,12,13,14]. Compared with MSSA, infections with MRSA have a higher morbidity and mortality rate [15] and are associated with higher treatment costs and longer hospital stays. Notably, vancomycin has been the first choice of clinical treatment for MRSA infections, but with its widespread use, the sensitivity of MRSA to vancomycin is decreasing, promoting the emergence of vancomycin-intermediate *S. aureus* (VISA) and vancomycin-resistant *S. aureus* (VRSA) [16,17]. Indeed, the high infection and mortality rate of *S. aureus*-resistant strains, as well as the increasingly severe drug resistance situation, has brought great challenges to clinical treatment, in turn presenting an urgent need to develop new drugs to combat those infections. However, it takes far longer to develop a new drug than it does for drug-resistant bacteria to spread—meaning it is important to find alternative effective countermeasures for the treatment of MRSA.

As a natural predator of bacteria, bacteriophages (phages) were initially used to treat staphylococcal skin infections in 1921 [18]. However, the rapid development of the antibiotic industry led to a decrease in phage usage. Now, as the current situation of drug resistance becomes more and more pressing, phages have again attracted our attention, although we still face many challenges when it comes to applying phages for clinical treatment [19]. While phages are generally considered to target specific bacterial species and leave the human host unaffected, phages have also recently been shown to affect the expression of mammalian genes and the pathophysiology of cancer cells in vitro [19,20]. While further research is required to validate such effects in relevant in vivo models, these findings warrant further investigations into the potential use of phages as an adjuvant to anticancer therapies. They also imply that phage therapy against MDR infections in cancer patients should be monitored closely for their potential effect on cancer progression.

Phages are remarkably species specific, therefore, phages selected for use in patients should target and be tested against clinical isolates from patients [21,22]. Therefore, a phage bank that contains well-characterized phages that target pathogens with a broad host range is critical to secure the success of personalized phage therapy. Those phages should be tested for stability, host range, and bacteriolytic activity to meet the requirements for clinical application. Additionally, only lytic phages can be used safely in the clinic. 

Therefore, the aim of this study was to isolate and characterize lytic anti-Staphylococcus phages towards the development of a phage cocktail to combat therapy refractory MSSA and MRSA infections.

## 2. Results

### 2.1. Phage Isolation 

A total of 193 nasal swabs, 5 soil samples, and 5 sheep feces samples were screened for the presence of phages. From those, four phages were isolated. APTC-SA-2 and APTC-SA-4 were isolated from 2 male patients with chronic rhinosinusitis without nasal polyps (CRSsNP) aged 60Y and 62Y respectively, after enrichment. The first patient had asthma and gastro-esophageal reflux disease (GORD); the second patient was also asthmatic and had diabetes mellitus. Both patients had positive *S. aureus* cultures. APTC-SA-12 was isolated from an environmental soil sample and APTC-SA-13 was isolated from sheep feces without enrichment. 

### 2.2. All Isolated Phages Had a Broad Host Range

The phages’ host range was investigated against 32 MSSA and 17 MRSA clinical isolates (all harvested from the sinonasal cavities of CRS patients) and 2 ATCC strains. 42/51 (82%) *S. aureus* strains were sensitive to APTC-SA-2; 44/51 (86%) *S. aureus* strains were sensitive to APTC-SA-4; 48/51(94%) *S. aureus* strains were sensitive or semi-sensitive to APTC-SA-12 and APTC-SA-13. From the 51 *S. aureus* strains tested, 18 were found to be resistant to at least one of the phages tested (35%). From those, only one strain (C100) was found to be resistant to all four phages and another one strain (C331) was found to be sensitive to only one of the phages tested. The remaining 16 strains were sensitive or semi-sensitive to at least 2/4 phages tested, indicating a high complementarity of 49/51 strains (96%). The combination of the four phages in a cocktail APTC-C-SA01 could lyse 50/51 (>98%) *S. aureus* strains when used at MOI = 0.1. Phage sensitivity for the four phages against all tested *S. aureus* strains is shown in Table 1.

### 2.3. The Phages Display Good Stability at Various pHs and Temperatures

**Figure 1:**
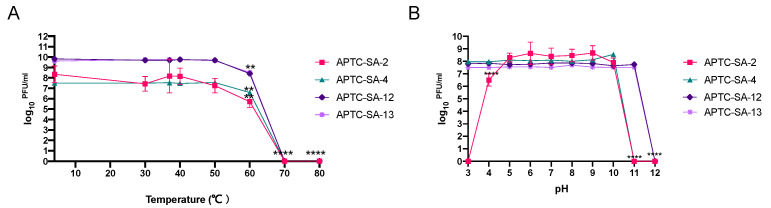
*S. aureus* phages’ temperature and pH stability tests. APTC-SA-2, APTC-SA-4, APTC-SA-12, and APTC-SA-13 phage stability testing measured in log_10_ ^PFU/mL^. (**A**) Phage stability at different temperatures (4, 30, 37, 40, 50, 60, 70, and 80 °C). (**B**) Phage stability at different pHs (3, 4, 5, 6, 7, 8, 9, 10, 11, and 12). The experiment was repeated 3 times. Bars represent standard deviations (SD). **, *p* < 0.01; ****, *p* < 0.0001.

Next, we determined the phages’ temperature (4 °C to 80 °C) and pH (3 to 12) stability. All four phages were stable at temperatures between 4 °C and 50 °C, with no significant reduction in phage titers observed. A significant reduction of phage titers was detected when incubated at 60 °C for all phages (<2 log10 PFU/mL) and at 70 °C and 80 °C, no phages were detected (Figure 1A). APTC-SA-2 was stable between pH 5 and pH 10. A significant reduction of viable phages was observed at pH 4, and no viable phages were observed at pH < 4 or pH ≥ 11. Phages APTC-SA-4, APTC-SA-12, and APTC-SA-13 were stable between pH 3 and pH 10 (APTC-SA-4) or pH 11 (APTC-SA-12 and APTC-SA-13) with a drop below detectable level in phage titers at pH 11 (APTC-SA-4) or pH 12 (APTC-SA-12 and APTC-SA-13) (Figure 1B).

### 2.4. Phage One-Step Growth Curve

**Figure 2:**
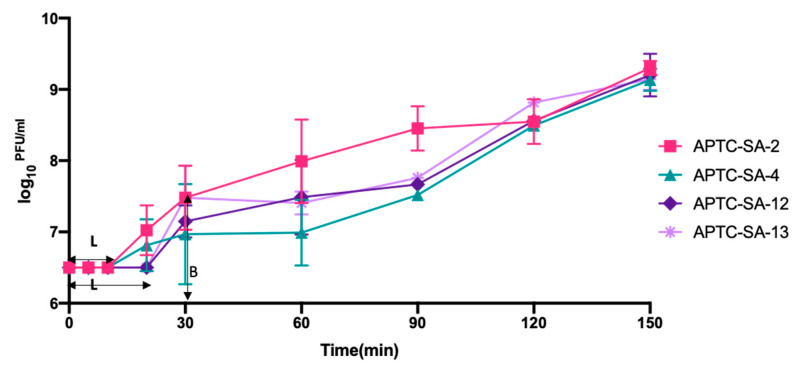
One-step growth curves of *S. aureus* phages. The log_10_ ^PFU/mL^ of phages in the cultures at different time points were tested. Each data point represents the mean from three independent experiments, and the error bars indicate SD. L = the latent period; B = burst size.

The latent periods and burst sizes of all phages were evaluated by performing a one-step growth curve. The latent periods of APTC-SA-2 and APTC-SA-4 were around 10 min and were shorter than the latent periods of around 20 min for ATPC-SA-12 and ATPC-SA-13. The burst sizes of the four phages were on average 30, 11, 14, and 18 PFUs/infected cell for APTC-SA-2, APTC-SA-4, ATPC-SA-12, and ATPC-SA-13, respectively (Figure 2).

### 2.5. Phage Inhibition Assay 

**Figure 3:**
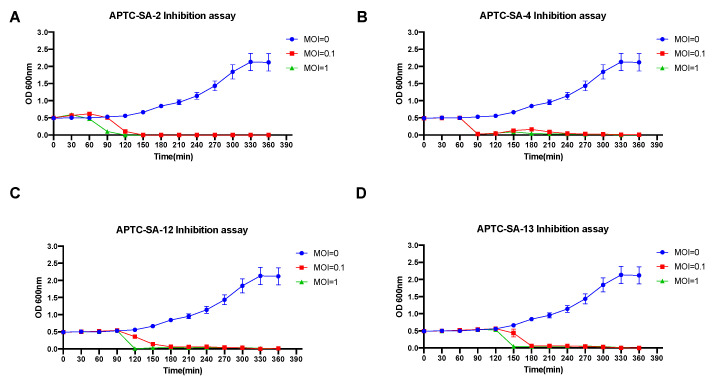
Inhibition assays of *S. aureus* phages. *S. aureus* ATCC25925 log-phase culture was infected with phages APTC-SA-2 (**A**), APTC-SA-4 (**B**), APTC-SA-12 (**C**), and APTC-SA-13 (**D**) at MOI = 0 (blue), 0.1 (red), and 1 (green). OD 600 nm was measured at time point = 0 and every 30 min up to time point 390 min. The error bars indicate SD from the results of three independent experiments.

*S. aureus* ATCC25923 was used to perform the in vitro phage inhibition assay with phage at MOI = 0.1 and 1, or MOI = 0 (no phage) as positive control. APTC-SA-2 at MOI = 1 and 0.1 reduced the bacterial growth at the 90 min and 120 min time points, respectively (Figure 3A), while ATPC-SA-4 reduced *S. aureus* growth at the 90 min time point for both MOI = 1 and MOI = 0.1 (*p* < 0.05) (Figure 3B). The effect of ATPC-SA-12 and ATPC-SA-13 on reducing the growth of *S. aureus* occurred at a later time point compared to APTC-SA-2 and APTC-SA-4, with APTC-SA-12 inhibiting the growth of *S. aureus* at 120 min (MOI = 1) and 180 min (MOI = 0.1), respectively (Figure 3C), and APTC-SA-13 at 150 min (MOI = 1) and 180 min (MOI = 0.1) (*p* < 0.05) (Figure 3D).

### 2.6. Phage Have Strong Bacteriolytic Activity against S. aureus Biofilm

**Figure 4:**
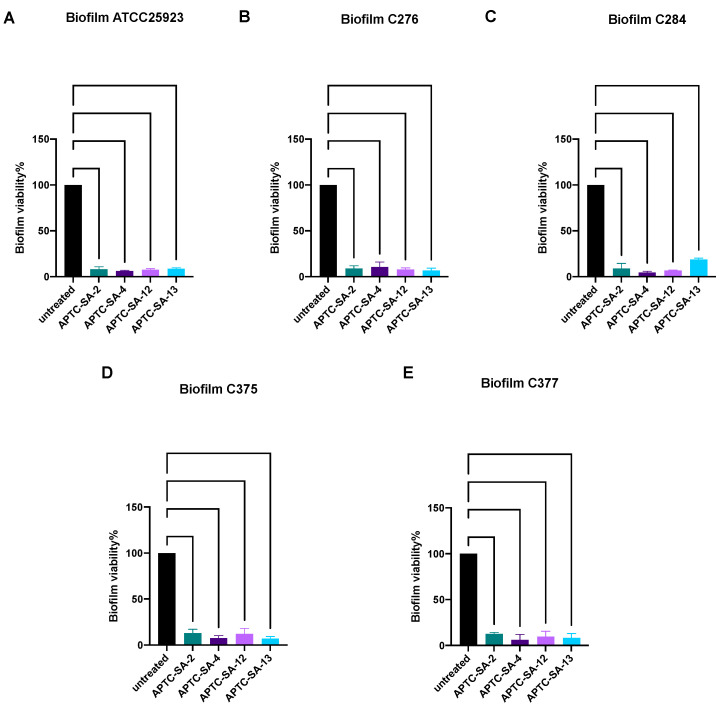
Biofilm assay. *S. aureus* biofilm (**A**) ATCC25923, (**B**) C276 (MSSA), (**C**) C284 (MSSA), (**D**) C375(MRSA), and (**E**) C377(MRSA) were treated with *S. aureus* phage (10^8^ PFU/mL) for 24 h followed by crystal violet assays to quantify the biofilm biomass. Significance was determined compared to untreated control. Data expressed as mean ± SD for three independent experiments.

Crystal violet assays were used to evaluate the various phages’ ability to reduce *S. aureus* biofilm biomass. *S. aureus* ATCC25923 and 4 clinical isolates (2 MSSA and 2 MRSA) were randomly selected to form biofilm and then treated with phage at 10^8^ PFU/mL for 24 h. All 4 tested phages could significantly reduce the biofilm biomass of all 5 *S. aureus* strains, compared to untreated control (Figure 4A–E), suggesting all four phages were able to reduce the biofilm biomass within 24 h in a single dosage treatment in vitro.

### 2.7. Phage Morphology Indicates Phages to Be Members of the Myoviridae

**Figure 5:**
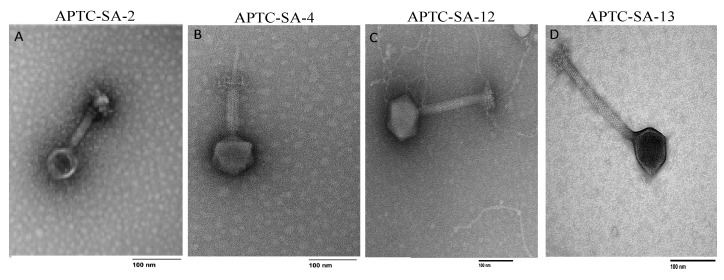
Morphology of Staphylococcus phages. Transmission electron microscopy images of *S. aureus* phage (**A**) APTC-SA-2, (**B**) APTC-SA-4, (**C**)APTC-SA-12, and (**D**) APTC-SA-13. Scale bar: 100 nm.

The morphology of the four *S. aureus* phages was investigated by TEM. The TEM images showed that all four phages had a polyhedral head and a long tail (Figure 5A–D), indicative of them belonging to the *Myoviridae* family according to the International Committee on Taxonomy of Viruses classification system.

### 2.8. Phage Genomic Analysis Indicates Lytic Nature and Absence of Virulence Genes 

**Figure 6:**
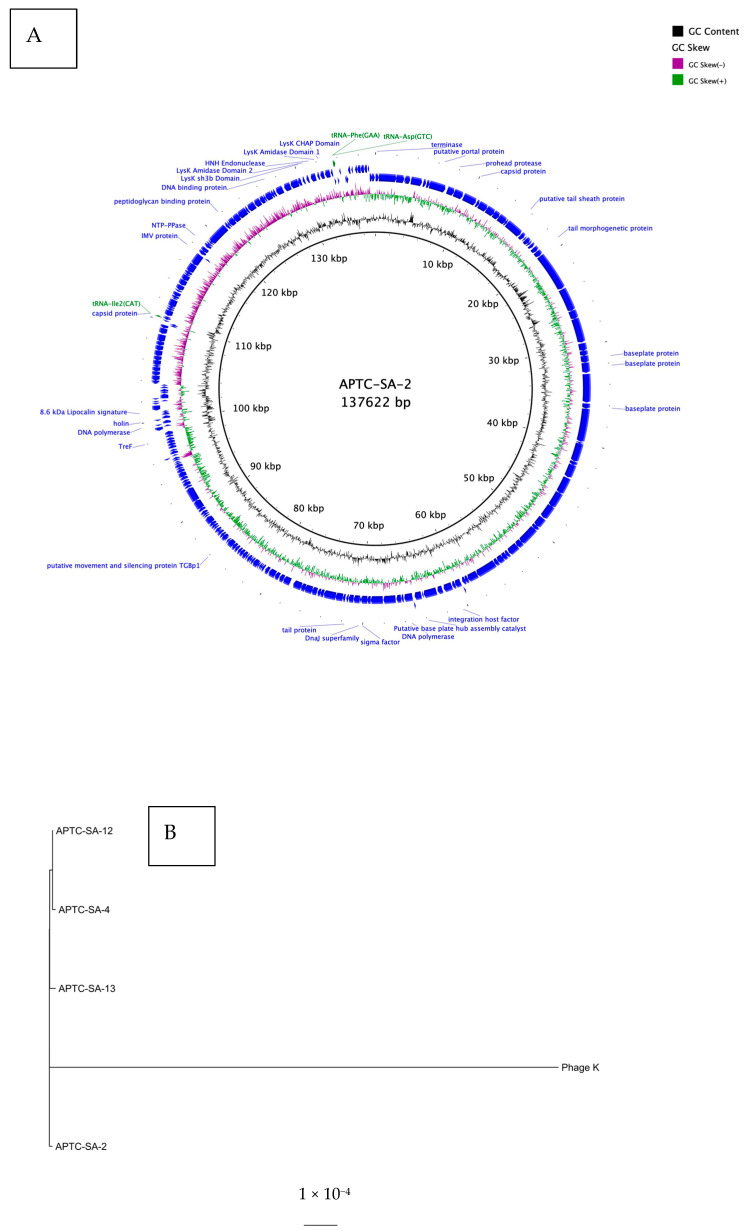
*S. aureus* phages. (**A**) Map of genomic organization of bacteriophage APTC-SA-2 shown as representative. The open reading frames with predicted annotations are indicated with blue arrows and predicted tRNAs are indicated with green arrows. (**B**) phytogenic tree of phages. A maximum likelihood phylogenetic tree analysis of APTC-SA-2, APTC-SA-4, APTC-SA-12, APTC-SA-13, and phage K was based on their nucleotide sequences.

Genomic analysis confirmed the four isolated phages to be of genus Kayvirus, subfamily Twortviridae, family Herelleviridae. The phages had a linear double stranded DNA with lengths of 137,622, 137,551, 138,214, and 141,041 bp with an average GC content of 30.42%, 30.41%, 30.43%, and 30.36% for APTC-SA-2, APTC-SA-4, APTC-SA-13, and APTC-SA-12, respectively. All genomes had between 245 and 250 predicted CDSs (Table 2; Figure 6A; Supplementary Figure S1). The phylogenetic analysis revealed significant mutational differences in the genomes of the 4 APTC-SA phages compared to the reference phage K genome. The genome of phage K differed from the APTC-SA phages at a rate of approximately 1.5 substitutions per 1000 bases (Figure 6B). The genomes of the APTC-SA phages differed between each other at a lower rate of approximate 1 substitution per 10,000 bases. None of the phages showed evidence of possessing putative lysogenic genes such as integrases. Additionally, all phages possessed the endolysin LysK. APTC-SA-12 lacks the phenylalanine tRNA tRNA-Phe(GAA), which is possessed by the other three phages. 

### 2.9. S. aureus Phages Show Good Complementarity with a Reduced Frequency of the Emergence of Bacteriophage-Insensitive Mutants (BIM) When Used in a Cocktail

We then determined the phage susceptibility of bacteriophage-insensitive mutants (BIMs) that were induced to be resistant to the four phages. Results show that BIMs induced to be resistant to any of the 4 phages were sensitive or showed intermediate sensitivity to at least 2/4 remaining phages.

We furthermore determined the frequency of bacteriophage-insensitive mutant (BIM) generation of the individual phages and of the phage cocktail APTC-C-SA01. Results showed the frequency of BIM generation was lower for APTC-C-SA01 compared to any of the single phages from the cocktail. Results are shown in Table 3A,B.

### 2.10. S. aureus Phages’ Activity against Various Species

The activity of the four phages alone and combined into the cocktail APTC-C-SA01 were further tested against various bacterial species. None of the phages could infect any of the non-Staphylococcal isolates (Table 4). However, from the 9 *Staphylococcus epidermidis* clinical isolates tested, 3/9 were sensitive to at least 1 of the phages and to the phage cocktail, 3/9 showed intermediate sensitivity, and 3/9 were insensitive or showed very weak sensitivity. Results are shown in Table 4.

## 3. Discussion

Here, four novel anti-staphylococcal phages were isolated from various sources, including patients’ nasal cavities, soil, and animal feces. All four phages were of the *Myoviridae* family and had lytic properties. They had a wide host range, lysing up to 94% of MSSA and MRSA clinical isolates and reference strains when used individually and up to 98% when combined. The phages were stable at a wide range of temperatures and pHs and were highly effective at reducing the biofilm biomass of 48 hr established *S. aureus* biofilms in vitro. The phages showed good complementarity and the frequency of BIM generation was reduced when phages were combined in a cocktail. Together, these properties support the potential for these novel phages to be used in cocktails to combat difficult-to-treat MSSA and MRSA infections. 

The increasing burden of infections with antibiotic-resistant pathogenic bacteria calls for the development of novel antimicrobial strategies. Specifically, infections with various multidrug-resistant (MDR) and virulent pathogens have been particularly concerning with the World Health Organization (WHO) calling for strategies to combat various pathogens including *Enterococcus faecium, Staphylococcus aureus, Klebsiella pneumoniae, Acinetobacter baumannii, Pseudomonas aeruginosa*, and *Enterobacter* spp. (ESKAPE pathogens). 

*S. aureus* is one of those ESKAPE pathogens and, while often considered a commensal species, it is also one of the leading causes of various infectious diseases. These can be mild, such as impetigo and skin and soft tissue infections, or they can be life threatening and cause pneumonia, osteomyelitis, endocarditis, and sepsis. *S. aureus* has also been associated with chronic relapsing infections such as chronic rhinosinusitis (CRS) [23]. The costs involved in treating *S. aureus* infections have increased significantly since 1998 [24], mainly due to the difficulty of treating infections with MRSA strains [25]. MRSA was first reported in 1961 [26], and due to the subsequent exponential mortality rate associated with it, it quickly became evident that a new antimicrobial approach was urgently needed.

Bacteriophages (phages) were considered an effective antimicrobial as early as the 1920s and have recently regained interest for their potential to treat infections with antibiotic-resistant strains including MRSA [27,28]. Phages have also been shown to affect the physiology of cancer cells in vitro [19,20] and have been shown to be effective against bacterial biofilms [29,30,31,32]. This latter is in line with our results where all MRSA strains tested in both planktonic and biofilm form were sensitive to at least one of the phages tested. Compared to antibiotics, phage therapy has several advantages. This includes the often high specificity to one bacterial species which minimizes the disruption of human commensals and normal microbiota [33], self-propagation of phages at the site of infection, and a good safety profile [28,34]. However, as with antibiotics, bacteria can evolve to become resistant to phages, rendering phage therapy ineffective [35]. For this reason, phage cocktails are preferred as bacteria would be less likely to develop resistance to the various phages present in the cocktail. This was also shown in this study where the emergence of BIMs in the presence of the phage cocktail was reduced approximately 20-fold compared to exposure to each of the individual phages. Combining various phages targeting the same species furthermore has the potential to widen the host range and thus their potential to be used in the clinical context. In this study, the host range of the individual phages ranged from 80% to 95% of the *S. aureus* isolates that were deemed to be sensitive while >98% of *S. aureus* were sensitive to the combination of all phages in a cocktail. In view of their complementary susceptibility profile, and the reduced frequency of emergence of BIMs in the presence of the cocktail, combining the four phages into the APTC-C-SA01 cocktail could indeed target almost all *S. aureus* clinical isolates with low frequency of BIM generation, suggesting that this cocktail could potentially be used for the majority of *S. aureus* and MRSA infections.

Furthermore, phages that are candidates for use in the clinical setting must be proven to be lytic and lack lysogenic genes that would allow them to integrate their genetic material into the bacterial host. Therapeutic phages must also be void of virulence and antimicrobial resistance genes [36,37]. Our results indicate the four novel phages to match those requirements. Their genomic and morphologic evaluation furthermore indicated they belong to the *Herelleviridae* family. This was formerly known as the *Myoviridae* family, which is the most common phage family used in the clinical setting but has been reclassified in 2018 [38,39]. Together, these results support their potential to be used in phage therapy applications.

Phage stability under storage conditions and at various temperature and pH is another challenge when it comes to delivering good-quality phages to the clinical setting. Our phages were shown to be highly stable at various temperatures and pH conditions, again supporting their potential for future therapeutic application. Further, a one-step growth curve was performed to determine the phage latency period and burst size. Our isolated phages showed a short latent (≤20 min) period and high burst size (≥11 PFU/infected cell), suggesting a result in favor of phage therapy [40]. 

In summary, here we report the isolation and characterization of four lytic anti-staphylococcal phages. The phages have a combined host range of >98% of MSSA and MRSA strains tested and had strong antibiofilm properties. The phages had a good stability and infectivity profile. The phages were able to complement each other and significantly reduced the frequency of BIM development when exposed to the phages combined in a cocktail. Together, these properties support their potential to be used in a cocktail in phage therapy applications.

## 4. Materials and Methods

### 4.1. Nasal Swab Collection and Identification of Bacteria

Ethics for the collection and use of clinical isolates was approved by the Central Adelaide Local Health Network Human Research Ethics Committee in Adelaide, South Australia (HREC/15/TQEH/132) and written informed consent was obtained from participants before collection of samples. Nasal swabs (International Medical Products, Brussels, Belgium) were applied within the sinonasal cavities of chronic rhinosinusitis (CRS) patients and were used to isolate *S. aureus* or phage. The diagnostic criteria for CRS with and without nasal polyps (CRSwNP and CRSsNP, respectively) were according to the European Position Statement (EPOS) on CRS guidelines [41].To isolate *S. aureus*, nasal swabs were vortexed in 100 uL Liquid Transport Medium, then transferred to Mannitol Salt Agar (MSA) plates (Oxoid, Thebarton, SA, Australia) and incubated at 37 °C overnight. Colonies were picked the next day and re-streaked on trypticase soy agar (Oxoid, Thebarton, SA, Australia) plates. The *S. aureus* identity was confirmed using MALDI-TOF system (Bruker, VIC, Australia) and isolates were stored in 25% glycerol in Tryptone Soya Broth (TSB) (Oxoid, Thebarton, Australia) at −80 °C until use. *S. aureus* strains were identified to be MRSA by an independent pathology laboratory (Adelaide Pathology Partners, Adelaide, SA, Australia).

### 4.2. Bacterial Strains and Growth Conditions

*S. aureus* frozen glycerol stocks were thawed and streaked on 1.5% TSA plates and incubated at 37 °C overnight. A single colony was picked and resuspended in 2 mL 0.9% saline to achieve McFarland (McF) = 0.5. 100 µL of resuspended bacterial solution was added into 10 mL trypticase soy broth (TSB) (Oxoid, Thebarton, SA, Australia) and incubated with shaking at 180 rpm for 24 h in a 37 °C incubator. Bacteria in the late logarithmic growth phase were used for further experiments. *S. aureus* ATCC25923, ATCC51650, and RN4220 were obtained from the American Type Culture Collection (ATCC, Manassas, VA, USA) and used as reference strains or the host strain for phage propagation. *Staphylococcus epidermidis* clinical isolates (*n* = 9, from CRS patients) were isolated by an independent pathology laboratory (Adelaide Pathology Partners, Adelaide, Australia). *Streptococcus pneumoniae* clinical isolates (*n* = 10) were donated by Dr Stephen Kidd, Molecular and Biomedical Science, The University of Adelaide, and *Pseudomonas aeruginosa* clinical isolates (*n* = 10) were donated by the Department of Otorhinolaryngology of the Academic Medical Centre, Amsterdam. *Acinetobacter baumannii* (ATCBAA1795), *Klebsiella pneumoniae* (ATCBAA1705; ATCBAA1904; ATC700721), *Escherichia coli* (ATCBAA2340 and ATCBAA2326), *Proteus mirabilis* (ATCBAA856), *Enterococcus faecalis* (ATC700802), and *Enterococcus faecium* (ATC51559) were from ATCC. 

### 4.3. Phage Isolation from Patient Nasal Swabs

Nasal swabs in 4 mL TSB were vortexed and the supernatant filtered through a 0.22 µm syringe filter (PALL Acrodisc, New York, NY, USA). Phages were then isolated using the conventional double-layer agar method (DLA) [42]. Briefly, 100 µL of filtered supernatant was incubated with 100 µL *S. aureus* RN4220 (1.5 × 10^8^ CFU/mL) overnight culture for 10 min at room temperature. The mixed culture was then combined with 4 mL 0.4% TSA and poured on top of 1.5% TSA plates. The plates were then incubated overnight at 37 °C to allow formation of plaques. 

### 4.4. Phage Isolation from Environmental Samples

Environmental samples (soil and sheep feces) were harvested and transferred to the lab on ice. Fresh samples were mixed with phage SM buffer (100 mM NaCl, 8 mM MgSO_4_, 50 mM Tris HCl (pH 7.5)) in 1:1 ratio (30 g environment sample in 30 mL SM buffer) and incubated for 1 h at 37 °C, with shaking at 180 rpm. The samples were then centrifuged at 13,000× rpm for 30 min. The supernatant was harvested and filtered using a 0.22 µm syringe filter (PALL Acrodisc, New York, NY, USA) for phage isolation using DLA as described. 

### 4.5. Phage Enrichment and Isolation

If no plaques were observed, enrichment experiments were performed. In brief, overnight *S. aureus* broth cultures were prepared followed by adding 100 µL bacterial cultures into 10 mL TSB and incubated at 37 °C, 180 rpm for 1 h. Then, 1 mL of filtered phage supernatant was added and cultured overnight at 37 °C with 180 rpm. The enriched sample was then centrifuged at 4000 rpm for 10 min to remove bacteria (Allegra X-30R Centrifuge, Beckman Coulter, NSW, Australia) and filtered using a 0.2 m syringe filter. DLA was performed to identify phage plaques as describe above. 

### 4.6. Phage Propagation and Phage Titer Determination

The purified phage was propagated using the RN4220 prophage free *S. aureus* strain. Then, 500 μL RN4220 overnight culture was incubated in 50 mL TSB for 1 h at 37 °C with 180 rpm agitation. Phage was then added at multiplicity of infection(MOI) = 1. After overnight incubation at 37 °C, 180 rpm, the supernatant was harvested after centrifugation (4000× *g*, 30 min) and filtration (0.2 μm syringe filter) and transferred into a new tube. To achieve high phage titers, phages were concentrated using a 100k MWCO Pierce^@^ Protein Concentrator PES (Thermo Scientific, Leicestershire, UK) before phage titration experiments. 

Phage titers were determined against *S. aureus* RN4220 using the double layer spot assay (DLSA). Briefly, 100 µL of *S. aureus* (RN4220, 1.5 × 10^8^ CFU/mL) overnight culture was mixed with 4 mL of 0.4% TSA and poured onto a 1.5% TSA plate. After 20 min, serially diluted samples of phage were spotted onto the double layer agar plates in 3 µL/spot in triplicates. Plaques were counted after overnight incubation at 37 °C, and phage titers were calculated based on the dilution.

### 4.7. Host Range/Sensitivity Test

Phage host range/sensitivity test was determined as described previously(Drilling, 2014 #2). Briefly, 200 μL of overnight *S. aureus* (1.5 × 10^8^ CFU/mL) culture was spread onto Columbian blood agar plates (Oxoid, Thebarton, SA, Australia) and plates were incubated at 37 °C for 1 h, then 5 μL phage (10^6^ PFU/mL) were spotted onto the plates in triplicates. Phage buffer solution (SM buffer and TSB) was spotted in the center of the plates as negative control. Phage sensitivity was determined as previously described after overnight incubation at 37 °C [43,44]. Confluent lysis zones observed on plates were defined as phage sensitive (+), semiconfluent lysis zones were defined as phage semi-sensitive (+/−), and no plaques were defined as phage insensitive(−) [44]. Host range was tested in triplicates.

### 4.8. Thermal and pH Stability

All thermal and pH stability tests were performed in triplicates. For thermal stability test, phages (10^9^ PFU/mL, in SM buffer) were incubated at different temperatures (40, 50, 60, 70, and 80 °C) for 1 h. For the pH stability, phages (10^9^ PFU/mL) were diluted 100-fold in SM buffer with different pH values (range from pH 3 to 12) and incubated for 1 h at room temperature. The phage titers were then determined using the DLSA.

### 4.9. One-Step Growth Curve

One-step growth curve was performed as detailed previously [45]. Briefly, 100 μL phages (1 × 10^8^ PFU/mL) were mixed with 1 mL *S. aureus* C369 (sensitive, 1.5 × 10^8^ CFU/mL) cultured at a MOI of 0.1 in 8.9 mL TSB and incubated at 37 °C for 5 min. The mixture was then centrifuged, and the pellets were resuspended in 9.9 mL fresh TSB. The resuspended pellets were incubated at 37 °C with shaking at 180 rpm and 100 μL mixture was taken every 30 min. The mixture was centrifuged at 13,000× *g* for 5 min, and the titers of phages were determined using the DLSA. The experiments were carried out in triplicates. The burst size was calculated by dividing the phage titers at post-burst plateau phase by the initial phage titers.

### 4.10. Inhibition Assay

Phages were cocultured with *S. aureus* ATCC 25923 and C377 overnight TSB cultures (5.5 × 10^8^ CFU/mL) at different MOIs (1 and 0.1) and incubated at 37 °C shaking at 180 rpm. *S. aureus* cultures and TSB were prepared alongside as a positive and a negative control, respectively. The absorbance (OD600) of the culture broth was measured every 30 min after the onset of incubation. The bacteriolytic activity was calculated at each measurement time point. The experiment was repeated 3 times.

### 4.11. Biofilm Assay

We diluted 1.0 McF unit of fresh *S. aureus* suspension in 0.9% saline 1:15 in NB and gently mixed it by inversion. An amount of 180 μL/well of the diluted suspension was added into a 96-well microtiter plate (BMG Labtech, Ortenberg, Germany) and incubated on a gyratory platform (Ratek, VIC, Australia) at 37 °C for 48 h to allow biofilm formation. Negative controls (NB only) were added to each plate. Biofilm was washed twice with PBS to remove planktonic bacteria followed by adding phage at a final concentration of 10^8^ PFU/mL for 24 h. Biofilm without phage treatment was set as positive control. Biofilm was then washed with PBS and stained with 0.1% crystal violet (Sigma-Aldrich, Castle Hill, NSW, Australia) for 15 min. The plate was rinsed with distilled water to remove extra crystal violet and air dried, then 30% acetic acid (Chem-Supply, Adelaide, SA, Australia) was added to elute the crystal violet staining. The absorbance was measured at 595 nm using the FLUOstar Optima microplate reader (BMG Labtech, Ortenberg, Germany) to quantify the biofilm biomass.

### 4.12. Transmission Electron Microscopy (TEM)

Transmission electron microscopy (TEM) was used to examine the phage morphology according to a published protocol with modifications [46]. Briefly, 5 μL of phage (>10^6^ PFU/mL) was placed on the coated side of a carbon/formvar coated grid (ProSciTech Pty Ltd., Townsville, QLD, Australia) for 3 min and dried with filter paper. Then, 5 µL of transmission electron microscopy (TEM) fixative (1.25% glutaraldehyde, 4% paraformaldehyde in phosphate buffer solution (PBS) and 4% sucrose) was added on the grid for 2 min and 5 µL of 2% uranyl acetate for 2 min in sequence. Fixed phages were then examined with FEI Tecnai G2 Spirit 120kV TEM (FEI Technologies Inc., Hillsboro, OR, USA).

### 4.13. Phage DNA Extraction and Sequencing

Phage DNA extraction was performed using the Phage DNA Isolation Kit (Norgen Biotek Crop, Thorold, ON, Canada) according to the manufacturer’s instructions. In brief, 500 μL of phage (10^7^~10^9^ PFU/mL) were treated with 10 μL (20 units) of Norgen’s RNase-Free DNase I (Norgen Biotek Crop, Thorold, ON, Canada) for 15 min at room temperature. DNase I was inactivated by incubation at 75 °C for 5 min, and 250 μL of lysis buffer was then added and the mixture vortexed for 10 s, followed by incubation at 65 °C for 15 min. Then, 160 μL of isopropanol was added to the lysate and mixed to precipitate DNA, and 650 μL of the solution was added to the column and centrifuged for 1 min at 6000× *g*. The column was washed with wash buffer and 40 μL of Elution Buffer B was applied to elute the DNA. DNA was stored at −80 °C before sequencing. Sequencing libraries were prepared using a modified protocol for the Nextera XT DNA library preparation kit with 150 bp paired end reads for all phages (Illumina Inc., San Diego, CA, USA). 

### 4.14. Genome Sequence Analysis

Quality checks were conducted on the raw fastq reads using FASTQC [47]. The raw fastq files were then trimmed using TrimGalore (https://github.com/FelixKrueger/TrimGalore (accessed on 10 February 2022)) and assembled into single contigs using Unicycler v 0.4.8 [48]. The assembled genomes were then annotated using MultiPhATE2 using PHANOTATE gene calls [49]. A multiple sequence alignment of the core genome of the phages, along with the genome of Phage K [50], was created using Roary v 3.13.0 [51] and MAFFT v 7489 [52]. Based on this alignment, a maximum-likelihood phylogenetic tree was created with the use of IQtree (v 2.0.4) [53]. Specifically, the resulting maximum likelihood tree was created using 1000 ultrafast bootstrap replicates, applying the SH-like approximate likelihood ratio test. The circular genome maps were created using BRIG v 0.95 [54].

### 4.15. Bacteriophage-Insensitive Mutants (BIM) Induction and Complementary Tests

Bacteriophage-insensitive mutant (BIM) strains for all 4 phages were isolated as described previously [55,56]. In brief, *S. aureus* C16 in exponential growth phase (OD 600 nm = 0.45, broth TSB) was treated with each phage at MOI = 100 for 10 min at room temperature (RT) and then mixed with 4 mL of 0.4% TSA. The mixture was poured on the top of 1.5% TSA and incubated at 37 °C for 24 h up to 48 h until BIMs appeared. Single BIM strains were picked, and the procedure was repeated at least 5 times. BIMs were stored in 50% glycerol in TSB at −80 °C or used in complementarity assays. In these, *S. aureus* C16 along with BIMs for each of the phages were tested for phage sensitivity to all tested phages using DLSAs as described. Confluent lysis zones observed on plates were defined as phage-sensitive (S), semiconfluent lysis zones were defined as phage-intermediate-resistant (I), and no plaques were defined as phage-resistant (R).

### 4.16. Determining the Frequency of Emergence of BIMs 

The frequency of BIM generation was determined by treating an overnight culture of *S. aureus* (approximately 10^9^ CFU/mL) with *S. aureus* phage alone or in a cocktail (APTC-C-SA01, containing APTC-SA-2, APTC-SA-4, APTC-SA-12, and APTC-SA-13 at a total PFU ratio of 1:1:1:1) at a MOI of 100. CaCl_2_ and MgSO_4_ were added to the mixture at final concentration of 10mM. Then, the mixture was incubated at 37 °C for 10 min and left to cool down at room temperature for 10 min. Serial dilutions were then plated (20 μL/spot) in triplicates on 1.5% TSA and incubated overnight at 37 °C. The colonies were counted and calculated. The BIM frequency was determined as surviving viable counts divided by the initial viable counts. All the experiments were performed three times.

### 4.17. S. aureus Phage Specificity Test

Phage specificity was performed using DLSAs as described above against various species, including *Staphylococcus epidermidis* clinical isolates (*n* = 9), *Streptococcus pneumoniae* clinical isolates (*n* = 10), *Pseudomonas aeruginosa* clinical isolates (*n* = 10), and various ATCC strains of *Acinetobacter baumannii*, *Klebsiella pneumoniae*, *Escherichia coli*, *Proteus mirabilis*, *Enterococcus faecalis,* and *Enterococcus faecium*. The experiment was repeated 6 times. Phage specificity was determined as previously described [45]. Confluent lysis zones observed on plates were defined as phage-sensitive (+), semiconfluent lysis zones were defined as phage-semi-sensitive (+/−), and no plaques were defined as phage-insensitive (−).

### 4.18. Statistical Analysis

GraphPad Prism (GraphPad Prism version 9.00; GraphPad Software, La Jolla, CA, USA) was used to graph and analyze statistical significance of data. Differences between groups were determined using a one-way analysis of variance (ANOVA). Significance was determined at a *p*-value < 0.05.

## Figures and Tables

**Table 1:** *S. aureus* phages’ host range.

ID	Strains		APTC-SA-2	APTC-SA-4	APTC-SA-12	APTC-SA-13
C16	***S. aureus***	**MSSA**	**+**	**+**	**+**	**+**
C19	***S. aureus***	**MSSA**	**+**	**+**	**+**	**+**
C24	***S. aureus***	**MRSA**	**−**	**+**	**+**	**+**
C25	***S. aureus***	**MSSA**	**+**	**+**	**+**	**+**
C52	***S. aureus***	**MSSA**	**+**	**+**	**+**	**+**
C64	***S. aureus***	**MSSA**	**+**	**+**	**+**	**+**
C72	***S. aureus***	**MSSA**	**+**	**+**	**+**	**+**
C79	***S. aureus***	**MSSA**	**+**	**+**	**+**	**+**
C100	***S. aureus***	**MSSA**	**−**	**−**	**−**	**−**
C148	***S. aureus***	**MRSA**	**+**	**+**	**+**	**+**
C154	***S. aureus***	**MSSA**	**+**	**+**	**+**	**+**
C183	***S. aureus***	**MSSA**	**+**	**+**	**+**	**+**
C194	***S. aureus***	**MSSA**	**+**	**+**	**+**	**+**
C209	***S. aureus***	**MRSA**	**−**	**+**	**+**	**+**
C222	***S. aureus***	**MRSA**	**+**	**−**	**+**	**+**
C246	***S. aureus***	**MSSA**	**+**	**+**	**+**	**+**
C256	***S. aureus***	**MSSA**	**+**	**+**	**+**	**+**
C263	***S. aureus***	**MRSA**	**−**	**+**	**+**	**+**
C273	***S. aureus***	**MSSA**	**−**	**+**	**+**	**+**
C274	***S. aureus***	**MSSA**	**+**	**+**	**+**	**−**
C275	***S. aureus***	**MSSA**	**−**	**+**	**+**	**+**
C276	***S. aureus***	**MSSA**	**+**	**+**	**+**	**+**
C278	***S. aureus***	**MSSA**	**+**	**+**	**+**	**+**
C283	***S. aureus***	**MSSA**	**+**	**+**	**+**	**+**
C284	***S. aureus***	**MSSA**	**+**	**+**	**+**	**+**
C285	***S. aureus***	**MSSA**	**+**	**−**	**−**	**+**
C286	***S. aureus***	**MSSA**	**+**	**−**	**+**	**+**
C295	***S. aureus***	**MRSA**	**+**	**+**	**+**	**+**
C297	***S. aureus***	**MSSA**	**+**	**+**	**+**	**+**
C298	***S. aureus***	**MSSA**	**+/−**	**+**	**+**	**+**
C311	***S. aureus***	**MSSA**	**+/−**	**+/−**	**+/−**	**+**
C318	***S. aureus***	**MSSA**	**+**	**+**	**+**	**+**
C322	***S. aureus***	**MSSA**	**+**	**+**	**+**	**+**
C324	***S. aureus***	**MSSA**	**+**	**+**	**+**	**+**
C329	***S. aureus***	**MSSA**	**+**	**+**	**+**	**+**
C331	***S. aureus***	**MRSA**	**−**	**+**	**−**	**−**
C342	***S. aureus***	**MRSA**	**−**	**+**	**+**	**+**
C355	***S. aureus***	**MSSA**	**+**	**+**	**+**	**+**
C363	***S. aureus***	**MSSA**	**+**	**+**	**+**	**+**
C364	***S. aureus***	**MSSA**	**+**	**−**	**+**	**+**
C369	***S. aureus***	**MRSA**	**+**	**+**	**+**	**+**
C370	***S. aureus***	**MRSA**	**+**	**+**	**+/−**	**+**
C371	***S. aureus***	**MRSA**	**+**	**−**	**+**	**+**
C373	***S. aureus***	**MRSA**	**+**	**−**	**+**	**+/−**
C374	***S. aureus***	**MRSA**	**+**	**+**	**+**	**+**
C375	***S. aureus***	**MRSA**	**+**	**+**	**+**	**+**
C376	***S. aureus***	**MRSA**	**−**	**+**	**+**	**+/−**
C377	***S. aureus***	**MRSA**	**+**	**+**	**+**	**+**
C378	***S. aureus***	**MRSA**	**+**	**+**	**+**	**+**
ATCC25923	***S. aureus***	**ATCC**	**+**	**+**	**+**	**+**
ATCC51650	***S. aureus***	**ATCC**	**+**	**+**	**+**	**+**
			**42/51** **(82%)**	**44/51** **(86%)**	**48/51** **(94%)**	**48/51** **(94%)**

MSSA = methicillin-susceptible *Staphylococcus aureus*; MRSA = methicillin-resistant *Staphylococcus aureus*; + = phage-sensitive; − = phage-resistant; +/− = phage-semi-sensitive. The experiment was repeated three times.

**Table 2:** Predicted functional ORFs of Staphylococcus phages.

Phage	Length (bp)	GC Percentage	Number of CDSs	Number of CDSs with Unknown Function	Number of CDSs with Known Function	Number of tRNAs	NCBI Accession Number
APTC-SA-2	137,622	30.42	246	213	33	3	OL960567
APTC-SA-4	137,551	30.41	245	210	35	3	OL960568
APTC-SA-12	141,041	30.36	250	214	36	2	OL960569
APTC-SA-13	138,214	30.43	247	213	34	3	OL960570

**Table 3:** (**A**) Activity of *S. aureus* phages against bacteriophage-insensitive mutants; (**B**) the frequency of the emergence of BIMs. C16: Parent strain; R1–16: C16 BIMs for APTC-SA-2, APTC-SA-6; APTC-SA-12 and APTC-SA-13; S: susceptible to the phage (clear plaque), I: intermediate sensitivity to the phage (turbid plaque), R: resistant to the phage (no plaque).

(A)				
	APTC-SA-2	APTC-SA-4	APTC-SA-12	APTC-SA-13
C16 Original	S	S	S	S
C16/APTC-SA-2 R1	R	S	S	I
C16/APTC-SA-2 R2	R	S	R	S
C16/APTC-SA-2 R3	I	S	S	S
C16/APTC-SA-2 R4	I	S	S	S
C16/APTC-SA-4 R5	R	R	S	S
C16/APTC-SA-4 R6	S	I	S	S
C16/APTC-SA-4 R7	I	R	S	S
C16/APTC-SA-4 R8	I	R	I	S
C16/APTC-SA-12 R9	S	S	R	S
C16/APTC-SA-12 R10	S	I	R	S
C16/APTC-SA-12 R11	S	S	R	I
C16/APTC-SA-12 R12	S	S	I	S
C16/APTC-SA-13 R13	S	S	I	R
C16/APTC-SA-13 R14	I	S	S	R
C16/APTC-SA-13 R15	S	R	S	I
C16/APTC-SA-13 R16	S	S	S	R
**(B)**				
**Phage**	* BIM Frequency± (Mean SD)			
APTC-SA-2	0.26 × 10^−7^		0.21 × 10^−7^	
APTC-SA-4	0.24 × 10^−7^		0.17 × 10^−7^	
APTC-SA-12	0.17 × 10^−7^		0.12 × 10^−7^	
APTC-SA-13	0.27 × 10^−7^		0.20 × 10^−7^	
APTC-C-SA01	0.01 × 10^−7^		0.009 × 10^−7^	

* Experiment performed using manufacturing strain C16. The average of three independent experiments is shown.

**Table 4:** *S. aureus* phage and the cocktail against non-target bacterial species.

Bacterial Test Strain			Phage Mix Sensitivity					
Genus	Species	ENT ID/ATCC ID	ATPC-SA-2	ATPC-SA-4	ATPC-SA-12	ATPC-SA-13	APTC-C-SA01	Control
*Staphylococcus*	*epidermidis*	C490	+/−	−	+/−	−	+/−	−
*Staphylococcus*	*epidermidis*	C498	−	−	−	−	−	−
*Staphylococcus*	*epidermidis*	C500	−	−	−	−	−	−
*Staphylococcus*	*epidermidis*	C503	+/−	−	+/−	−	+/−	−
*Staphylococcus*	*epidermidis*	C520	−	−	−	−	−	−
*Staphylococcus*	*epidermidis*	C525	+	+/−	+	+	+	−
*Staphylococcus*	*epidermidis*	C526	+	+/−	+	+	+	−
*Staphylococcus*	*epidermidis*	C529	+/−	−	+/−	+/−	+/−	−
*Staphylococcus*	*epidermidis*	C536	+/−	+/−	+	+	+	−
*Streptococcus*	*pneumoniae*	C819	−	−	−	−	−	−
*Streptococcus*	*pneumoniae*	C820	−	−	−	−	−	−
*Streptococcus*	*pneumoniae*	C821	−	−	−	−	−	−
*Streptococcus*	*pneumoniae*	C822	−	−	−	−	−	−
*Streptococcus*	*pneumoniae*	C823	−	−	−	−	−	−
*Streptococcus*	*pneumoniae*	C824	−	−	−	−	−	−
*Streptococcus*	*pneumoniae*	C825	−	−	−	−	−	−
*Streptococcus*	*pneumoniae*	C826	−	−	−	−	−	−
*Streptococcus*	*pneumoniae*	C827	−	−	−	−	−	−
*Streptococcus*	*pneumoniae*	C828	−	−	−	−	−	−
*Pseudomonas*	*aeruginosa*	C401	−	−	−	−	−	−
*Pseudomonas*	*aeruginosa*	C403	−	−	−	−	−	−
*Pseudomonas*	*aeruginosa*	C405	−	−	−	−	−	−
*Pseudomonas*	*aeruginosa*	C406	−	−	−	−	−	−
*Pseudomonas*	*aeruginosa*	C407	−	−	−	−	−	−
*Pseudomonas*	*aeruginosa*	C408	−	−	−	−	−	−
*Pseudomonas*	*aeruginosa*	C410	−	−	−	−	−	−
*Pseudomonas*	*aeruginosa*	C412	−	−	−	−	−	−
*Pseudomonas*	*aeruginosa*	C413	−	−	−	−	−	−
*Pseudomonas*	*aeruginosa*	C414	−	−	−	−	−	−
*Acinetobacter*	*baumannii*	ATCBAA1795	−	−	−	−	−	−
*Klebsiella*	*pneumoniae*	ATCBAA1705	−	−	−	−	−	−
*Klebsiella*	*pneumoniae*	ATCBAA1904	−	−	−	−	−	−
*Klebsiella*	*pneumoniae*	ATC700721	−	−	−	−	−	−
*Escherichia*	*coli*	ATCBAA2340	−	−	−	−	−	−
*Escherichia*	*coli*	ATCBAA2326	−	−	−	−	−	−
*Proteus*	*mirabilis*	ATCBAA856	−	−	−	−	−	−
*Enterococcus*	*faecalis*	ATC700802	−	−	−	−	−	−
*Enterococcus*	*faecium*	ATC51559	−	−	−	−	−	−
Cell Color	Interpretation							
	No plaque (No activity)							
	Semiconfluent lysis zones (partial activity)							
	Confluent lysis zones (full activity)							

## Data Availability

Not applicable.

## Supplementary Materials

The following supporting information can be downloaded at: https://www.mdpi.com/article/10.3390/ijms23116116/s1.
