# Supplementary material for: APTC-C-SA01: A Novel Bacteriophage Cocktail Targeting Staphylococcus aureus and MRSA Biofilms
Source: Int J Mol Sci. 2022 May 30;23(11):6116. doi: 10.3390/ijms23116116 (PMC9181636; doi:10.3390/ijms23116116)
Supplement: Supplementary file 1 [file ijms-23-06116-s001.zip › ijms-1721308-supplementary.pdf]

International Journal of *Molecular Sciences*

MDPI

Supplementary Figure 1

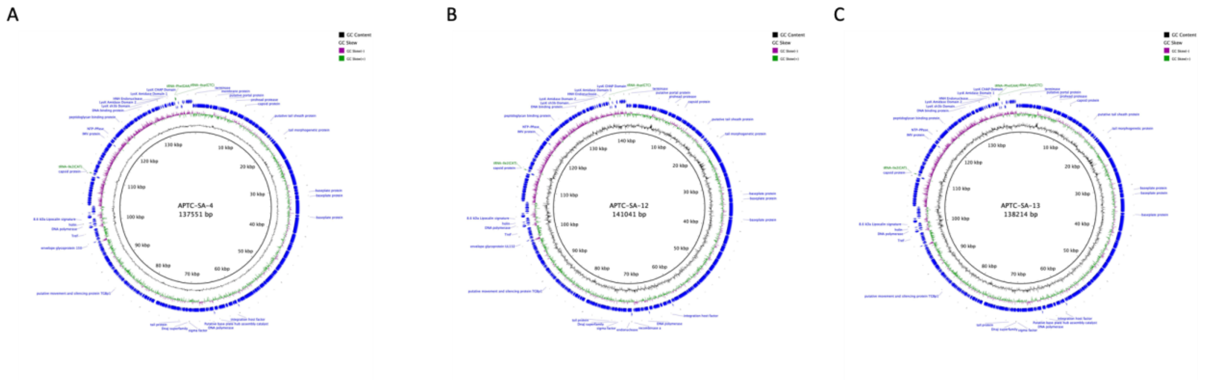

Supplementary Figure 1: Map of genomic organization of bacteriophage (A) APTC-SA-4; (B) APTC-SA-12 and (C) APTC-SA-13. The Open Reading Frames with predicted annotations are indicated with blue arrows and predicted tRNAs are indicated with green arrows.
